# The cost‐of‐living crisis is feeding the paradox of obesity and food insecurities in the UK

**DOI:** 10.1002/oby.23740

**Published:** 2023-04-05

**Authors:** Alexandra Johnstone, Marta Lonnie, the FIO‐Food project team

**Affiliations:** ^1^ The Rowett Institute, School of Medicine, Medical Sciences and Nutrition University of Aberdeen Aberdeen Scotland UK

## Abstract

Interconnections between the cost of living crisis and health inequality.

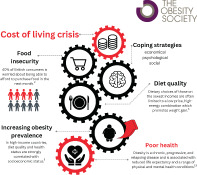

The cost‐of‐living crisis, with increasing food and energy costs [1], will impact the obesity epidemic in the UK, producing more diet and health inequalities for those living with obesity. In September 2022, 40% of British consumers reported that they were worried about being able to afford to purchase food in the next month [2]. In high‐income countries, diet quality and health status are strongly correlated with socioeconomic status [3]. In Scotland, obesity rates are 36% in areas in the highest quintile of deprivation (based on the Scottish Index of Multiple Deprivation) and 26% in areas in the lowest social deprivation quintile [4]. With slight regional variations, around two thirds of adults in the UK are living with overweight or obesity [5].

Food insecurity (FI) is defined as “the lack of secure access to sufficient amounts of safe and nutritious food for normal growth and development and an active and healthy life” [6]. Just as hunger can be experienced by food‐secure individuals, FI can be experienced without hunger [7]. For those living with obesity, the rising cost of healthier food creates a food‐insecure environment, not related to access to food, but rather, access to affordable and healthier food. This is a multifactorial phenomenon that encompasses qualitative, psychological, and social dimensions [7]. The scale of the problem is dynamic, with a sharp increase in FI since the COVID‐19 pandemic. In September 2022, 25% of households with children were reported as being food insecure [8]. This is a staggering 2.5‐fold increase in the number of households experiencing FI since January 2021 [8]. For families on low income, the poorest fifth of the UK population need to spend 47% of their disposable income to consume a healthy diet according to the Eatwell Guide, in contrast to 11% needed by the richest fifth in the UK [9]. The supermarket food environment has an important role to play in the mediation of better food purchasing for consumers, including those living with obesity.

The current food system in the UK enables the consumption of highly processed foods, high in energy density, fat, sugar, and salt, which are cheaper than more nutritious foods. In the UK, healthier foods are three times more expensive per calorie than unhealthy foods [9], with a similar trend observed in the United States [10]. It has been shown that food sources of protein, fiber, vitamins, and minerals cost more per 100 g, after adjustment for energy [10]. As a result, the dietary choices of those on the lowest incomes are often limited to a low‐price, high‐energy combination, which, in the long term, can promote weight gain, especially when combined with a sedentary lifestyle [3]. It is an apparent paradox that along with the increasing rates of FI, an increasing prevalence of obesity is observed—a link that may seem self‐contradictory to the general public [11]. However, the intersection between low income and obesity is more complex than simply the “energy in–energy out” concept and is not easy to reconcile. Social bias and discrimination experienced by people living with obesity and FI mean that the orthodox solution of “eating less” and “exercising more” is a repressive solution [12]. We need evidence‐based solutions to support the National Food Strategy (2021) approach to “deliver safe, healthy, affordable food, regardless of where people live or how much they earn” [13].

What is the true cost of this status quo? Poor diet is the primary risk factor for cardiovascular diseases, type 2 diabetes, and some forms of cancers and it has a profound effect on all‐cause disability‐adjusted life years [14]. At the national level, it is estimated that by 2050, the NHS costs related to overweight and obesity will reach £9.7 billion; a £3.6 billion increase from 2014/2015 [5]. The anticipated abandonment of the Department of Health's Obesity Strategy [15] by the UK government is shortsighted and it will likely widen the existing health inequality gap even further, with the potential to worsen the health of the public and increase the prevalence of obesity in both adults and children.

As scientists, we pledge to address this problem head on [16]. In the FIO‐Food (Food Insecurity in people living with Obesity) project [17], we aim to provide actionable evidence for responsive policy on retail strategies to address dietary inequalities in people living with obesity and food insecurity. As research shows, structural changes in the food system are needed, as behavioral interventions appear to have little impact on tackling FI among low‐income families [18]. Over the next 3 years, our goal is to identify how we can provide realistic and robust solutions while paying close attention to the cost‐of‐living crisis. Only the combined efforts and coproduction of systems‐wide changes, driven by stakeholders and those living with obesity and FI, have real transformative potential to make the food environment healthier, more sustainable, and affordable, for all.

## AUTHOR CONTRIBUTIONS

AJ and ML conceived the idea for the article. Both authors were involved in writing the paper and had final approval of the submitted and published versions.

## FUNDING INFORMATION

The Biotechnology and Biological Sciences Research Council (BBSRC) funded this reasearch, Grant Award BB/W018020/1, for FIO Food: Food Insecurity in people living with Obesity ‐ improving sustainable and healthier food choices in the retail FOOD environment.

## CONFLICT OF INTEREST STATEMENT

The authors declared no conflict of interest.
